# Impact of live music therapy compared with storytelling on maternal depression and psychological distress in mothers of preterm infants: a secondary analysis of a randomized controlled trial

**DOI:** 10.1186/s40748-026-00300-8

**Published:** 2026-10-01

**Authors:** Susann Kobus, Nina Jacqueline Klasen, Alisa Weinkoetz, Felicia Schneider, Ursula Felderhoff-Mueser, Nora Bruns

**Affiliations:** 1 Department of Pediatrics I, University Hospital, University of Duisburg-Essen, Hufeland Street 55, 45147 Essen, Germany; 2 Center for Translational Neuro- and Behavioral Sciences, Faculty of Medicine, C-TNBS, University Duisburg-Essen, Essen, Germany; 3 Center of Artistic Therapy, University Medicine Essen, Essen, Germany; 4 Alanus University of Arts and Social Sciences, Alfter, Germany; 5 Research Institute of Creative Arts Therapies, Alanus University, Alfter, Germany

**Keywords:** Music therapy, Preterm infants, Neonatal intensive care unit, Storytelling, Neonatology, Maternal depression, Psychological distress

## Abstract

**Introduction:**

Music therapy in neonatology is becoming increasingly important and is now considered an evidence-based intervention that effectively complements standard medical care for preterm infants and their mothers in accordance with family-centered care. This study aimed to compare the effects of live-performed music therapy (MT) and storytelling (ST) in the neonatal intensive care unit (NICU) on psychological distress and depression among mothers of preterm infants.

**Methods:**

The study was conducted as a secondary analysis of a randomized controlled trial (RCT), enrolling 60 preterm infants born between 32+0 to 36+6 weeks of gestation. Infants were block-randomized 1:1 to receive either MT or ST. Both interventions were delivered live to the preterm infants three to four times per week from birth until hospital discharge from hospital. The degree of depressive symptoms, anxiety and acute stress reactions of their mothers were measured using the German version of the Center for Epidemiologic Studies Depression Scale (CES-D) and the Impact of Event Scale–Revised (IES-R). Assessments were conducted on the first day of delivery prior to randomization (Timepoint 1 [T1]), at infants discharge from hospital (Timepoint 2 [T2]) and around the infants’ first birthday (Timepoint 3 [T3]).

**Results:**

The infants of 30 mothers [mean age 33.8 ± 6.1 years (range 21–47)] received MT and the infants of an additional 30 mothers [mean age 32.9 ± 5.3 years (range 21–45)] received ST. A total of 552 sessions were conducted, including 303 live music therapy sessions with a mean duration of 24 ± 2.5 minutes and 249 live storytelling sessions with a mean duration of 23 ± 4.1 minutes. Mothers whose infants received music therapy demonstrated greater reductions of depressive and stress symptoms between T1 and T2 compared to mothers whose infants received storytelling (MT: CES-D mean difference in total score –22.4, IES-R mean difference of total score –3.9; ST: mean difference in CES-D total score –10.7, mean difference of IES-R total score –1.1). Effect sizes (Cohen’s d) for the differences between T1 and T2 were large for total CES-D (1.0) and total IES-R (1.5), but weak for the differences between T1 and T3 (total CES-D 0.4, total IES-R 0.4). The absolute values at T3 did not differ as much as those at T2 between the two groups, but remained slightly more favorable in mothers whose infants received MT (mean total CES-D 6.7, 95% Confidence Interval (CI) 5.9–7.6; mean total IES-R –1.8, 95% CI –2.2–(–1.4)) than in mothers whose infants received ST (mean total CES-D 10.8, 95% CI 9.1–12.4; mean total IES-R –1.7, 95% CI –1.9–(–1.2)).

**Discussion:**

These findings indicate that mothers of preterm infants may benefit from music therapy as a supportive, non-medical intervention in alleviating psychological symptoms and distress during their infants’ NICU stay and may continue to support maternal well-being in the longer term, up to the infants' first birthday.

## Introduction

More than one in ten infants worldwide is born before completing 37 weeks of gestation, resulting in approximately 15 million preterm births each year [1]. Preterm birth represents a leading cause of neonatal morbidity and mortality globally [2]. In recent years, advances in perinatal care have substantially increased the survival rates of preterm infants [3].

Pregnancy and the transition to motherhood constitute a complex developmental process, encompassing profound biological, emotional, cognitive, and social changes, beginning during pregnancy and continuing into the postpartum period. This process has been described as maternal role attainment and was later reconceptualized as “becoming a mother” [4, 5].

The transition to motherhood initiates the mother–child relationship and represents a critical period of family adjustment, involving significant changes in maternal lifestyle and daily routines [6]. Childbirth itself represents a potential risk factor for the development of post-traumatic stress symptoms, anxiety, and depression in women [2, 7–11]. These challenges are further intensified for mothers of preterm infants, who often face limited opportunities for early contact due to their infant’s hospitalization in the NICU [7–9, 12]. Admission to the NICU is frequently experienced as traumatic, and is characterized by uncertainty regarding infant survival, exposure to complex medical technology, and disruption of expected maternal roles. Parental role alteration, infant appearance, and medical procedures have been consistently identified as the strongest stressors [13, 14].

Besides these acute stressors, preterm delivery abruptly interrupts pregnancy before the completion of the mother’s prenatal psychological adjustment. The anticipated timeline for mental preparation is compressed, and mothers may feel unprepared for immediate caregiving responsibilities in a highly medicalized neonatal environment. Research shows that mothers of preterm infants frequently report difficulties in feeling like a “real mother” during early hospitalization, particularly when physical separation from the infant occurs [15]. Consequently, mothers of preterm infants are at elevated risk for postpartum depression, anxiety disorders, and post-traumatic stress disorder (PTSD) [16–18]. Prospective studies report that over 70% experience clinically relevant symptoms of trauma, anxiety, or depression in the first weeks postpartum [19], with systematic reviews estimating that 25–40% develop significant depressive or PTSD symptoms following NICU hospitalization [20]. Psychological distress may persist up to 12 months postpartum and beyond, particularly following extreme preterm birth [21, 22].

Early mother–infant separation and limited caregiving opportunities in the NICU contribute to heightened helplessness, emotional distancing, and reduced perceived maternal control, impairing maternal role attainment and the consolidation of maternal identity [23]. Maternal distress has been linked to altered infant neurobehavioral functioning, suboptimal developmental trajectories, and reduced maternal sensitivity, potentially affecting long-term child outcomes [24–26]. External stressors, such as the COVID-19 pandemic, can further increase maternal burden [27, 28], and broader population studies suggest that preterm delivery, including moderate and late preterm births, is associated with a higher long-term risk of maternal mental health disorders [29]. Physical separation, the infant’s medical condition, and distinct preterm characteristics such as very low birth weight, reduced muscle tone, and immature organ functioning, significantly influence maternal emotional responses and early bonding [14, 30, 31]. Following NICU admission, mothers often perceive a temporary relinquishment of caregiving responsibilities to medical staff, leading to reduced perceived competence, increased stress, and feelings of responsibility or guilt regarding preterm birth [32–36].

Accordingly, there is a clear need to identify interventions that effectively mitigate stress-related symptoms in mothers of preterm infants, while simultaneously fostering the development of a healthy mother–infant relationship [37]. Music therapy may be particularly well suited to this context because it combines emotional, relational, and sensory experiences within a flexible, family-centred intervention. Research indicates that structured approaches such as music therapy may assist mothers of preterm infants in developing supportive caregiving behaviors that positively influence both maternal well-being and infant outcomes [38]. As a personalized and family-centred modality [39], music therapy has demonstrated a wide range of beneficial effects for both preterm infants and their parents [40]. In addition, music therapy serves as a stress-reducing intervention, contributing not only to the short-term physiological stabilization of preterm infants [41] but also to improvements in maternal emotional states and behavioral responses [42–49]. Within neonatal intensive care settings, evidence also suggests that music therapy can alleviate symptoms of anxiety and depression among parents during hospitalization [50]. Importantly, the therapeutic target and format of music therapy vary across studies. Some interventions primarily involve the infant, with the mother present and participating to different degrees, whereas others focus more directly on the mother or explicitly target the mother–infant dyad. This distinction is relevant because interventions involving active maternal participation may influence maternal psychological outcomes through mechanisms related to self-efficacy, emotional regulation, and parent–infant bonding, in addition to any direct effects on the infant. Overall, parents tend to perceive music therapy as a meaningful and supportive intervention that helps them manage the demands and emotional challenges associated with the early stages of their infant’s NICU stay [41].

To distinguish intervention-specific effects of music therapy from nonspecific benefits of therapeutic attention and interpersonal support, storytelling was selected as an active comparator. Storytelling may represent a relevant psychosocial intervention in the neonatal setting because, similar to music therapy, it provides structured, individualized, and professionally guided interaction while requiring active engagement with the parent–infant dyad. An active comparator was selected because a comparison with usual care or no additional intervention would not allow the effects of music therapy to be readily distinguished from nonspecific effects of receiving an additional therapeutic intervention. Both interventions provide structured, professionally guided interaction and opportunities for emotional expression, communication, and relational engagement. Thus, storytelling controls, at least in part, factors such as professional attention, social support, and participation in a structured therapeutic activity that may contribute to improvements in maternal well-being. The use of an active comparator is particularly relevant in neonatal care, where individualized caregiving and supportive interpersonal interactions may themselves influence parental stress and well-being [39, 51]. Recent randomized evidence comparing live-performed music with storytelling in preterm infants likewise supports the use of storytelling as a caregiving comparator, demonstrating beneficial effects of both interventions while allowing the intervention-specific effects of music to be examined [52]. At the same time, storytelling does not incorporate the specific musical, rhythmic, and multisensory elements inherent in live music therapy. Comparing music therapy with an active psychosocial intervention therefore provides a more stringent test of whether observed effects are attributable to music therapy-specific mechanisms rather than to therapeutic attention alone [53].

This study aimed to examine the prevalence of maternal psychological distress and well-being among mothers of preterm infants born at or after 32 weeks of gestation. Maternal outcomes were compared between mothers of infants who received music therapy and those who received storytelling at three timepoints: directly after birth before randomization, at the infant’s discharge from the hospital, and around the infant’s first birthday. We hypothesized that exposure to music therapy would reduce maternal distress and improve psychological well-being.

## Materials and methods

### Study design

The present study is a secondary analysis of prespecified maternal outcomes from a prospective, randomized, controlled clinical trial with two parallel study arms. The parent randomized controlled trial investigated the effects of live-performed music therapy versus storytelling on physiological and behavioral parameters in preterm infants and has been reported previously by Weinkoetz et al. [52]. The present report focuses on maternal psychological outcomes, specifically depressive symptoms and psychological distress, which were assessed using standardized maternal questionnaires.

In the parent trial, a total of 60 preterm infants were recruited and randomized 1:1 to either the music therapy (MT) or storytelling (ST) group. Block randomization was chosen to minimize the risk of unequal distribution of participants between the groups and to enhance the internal validity and comparability of the study arms. Randomization was performed in fixed blocks of 20 participants, thereby maintaining allocation balance while reducing predictability. Infants in the intervention group received standard neonatal care plus live-performed music therapy, whereas infants in the control group received standard neonatal care plus storytelling.

The present secondary analysis uses the maternal outcome data collected within this randomized trial. These outcomes are distinct from the infant physiological and behavioral outcomes reported in the companion publication by Weinkoetz et al. [52].

### Sample size

The sample size for the parent randomized controlled trial was determined based on the primary infant outcomes. The maternal psychological outcomes assessed in the present study, including depressive symptoms and psychological distress, were prespecified secondary outcomes of the parent trial. Therefore, the sample size was not independently determined or powered specifically for the maternal outcomes analyzed in the present secondary analysis.

An a priori sample size calculation was performed to determine the number of infants required to detect a clinically meaningful difference in maternal psychological distress between the two groups. Based on this analysis, a minimum total sample size of 54 mothers was estimated to be necessary. The calculation was informed by prior data [54] and assumed a standardized effect size of Cohen’s *d* = 0.6 for the primary outcome, a two-sided significance level (α) of 0.05, and a statistical power of 70% (1 − β = 0.70).

A total of 60 infants were recruited and randomized, with 30 participants allocated to each study arm. The present analyses of maternal psychological outcomes were conducted using the available data from this prespecified secondary outcome assessment and should therefore be considered exploratory with respect to statistical power for these endpoints.

Given the inherent uncertainty in precisely estimating the true effect size in advance, as well as the potential for participant attrition during the study period, the planned recruitment target was increased beyond the minimum required sample. Accordingly, the final target enrollment was set at 60 participants, with 30 allocated to each study arm to maintain adequate statistical power in the event of dropouts or protocol deviations.

### Eligibility and recruitment

The study took place in the neonatal care unit and neonatal intensive care unit of the University Hospital Essen, Germany, a tertiary referral center providing specialized care for preterm and high-risk neonates. Mothers of preterm infants born at this hospital between April 2023 and January 2024 with a gestational age (GA) of 32 + 0 to 36 + 6 weeks were eligible for participation.

Prior to study enrollment, parents received written and verbal information about the study’s objectives, procedures, potential risks and benefits, and the voluntary nature of participation. In addition, the same documents also included written consent for data collection and processing in accordance with applicable data protection regulations. Parents received the information sheet and a copy of the signed consent form. The original signed consent forms will be retained for a maximum of 10 years following study completion.

This study was conducted in accordance with established ethical standards for research involving human participants. The study protocol was reviewed and approved by the local ethics committee, the Approval Committee of the Medical Faculty of the University of Duisburg-Essen (reference number 23-11159-BO) on April 23, 2023, prior to the commencement of the study. The trial was registered retrospectively in the German Clinical Trials Registry (DRKS00036274) on February 25, 2025. All study procedures were conducted in accordance with the principles of the Declaration of Helsinki and adhered to the Consolidated Standards of Reporting Trials (CONSORT) guidelines to ensure methodological rigor and transparent reporting. Informed consent was obtained from all individual participants included in the study. Human Ethics and Consent to Participate declarations.

### Interventions

#### Music therapy intervention

Participants infants allocated to the music therapy (MT) group received live, individualized music therapy sessions with a frequency of approximately three to four sessions per week. Each session had a mean duration of about 25 min, allowing sufficient time for therapeutic engagement while respecting the physiological vulnerability and limited tolerance of preterm infants. The intervention was initiated after randomization and continued until the infant’s discharge from the neonatal unit or transfer to another ward.

The music therapy sessions followed a standardized protocol, and intervention integrity was monitored. All sessions were conducted as individualized one-on-one interventions by a certified music therapist with specialized training in neonatal music therapy and two trained medical students. The therapeutic approach was tailored to the infant’s behavioral state, physiological stability, and individual cues, with continuous monitoring of tolerance and responsiveness throughout each session.

In cases involving multiple births (e.g., twins or triplets) who were accommodated side by side in a shared bed and randomized to the same intervention group, the therapy was administered simultaneously. Care was taken to maintain an equal spatial distance between the therapist and each infant to ensure balanced auditory exposure. The orientation of the instrument was alternated between infants during the session to provide equivalent acoustic stimulation and minimize potential bias in sensory input.

The instrument employed in this study was the Sansula (Hokema Kalimbas, Germany), a commercially manufactured modified Kalimba (thumb piano) featuring a wooden soundboard with metal tines mounted on a drum membrane to enhance resonance. This construction produces a particularly soft, warm, and sustained tonal quality characterized by gentle overtones and low dynamic intensity, making it especially suitable for use in the sensitive acoustic environment of neonatal care settings.

The instrument is held with both hands and played by plucking the tines with the thumbs, generating continuous, flowing tones with a calm and resonant timbre. For this study, the instrument Sansula tuned to A minor was used to provide a harmonically coherent and emotionally neutral tonal framework. Predominantly, the three lowest tones (A, C’, and E’) were utilized, as lower frequencies are perceived as less stimulating and more soothing, thereby minimizing the risk of overstimulation in preterm infants. The musical material was not pre-composed but improvised in real time. This improvisational approach allowed the therapist to responsively adapt melodic contour, tempo, rhythm, phrasing, and dynamics to the infant’s behavioral state and physiological parameters, such as breathing pattern, facial expression, motor activity, and overall arousal level. In this way, the infant’s cues actively shaped the musical interaction, fostering a process of attuned, individualized therapeutic engagement.

During the intervention, infants remained either in their incubator, in a heated cot, or were held by a parent or relative depending on their clinical condition and routine care procedures. The therapist positioned herself either standing or seated beside the infant, maintaining approximately 20–30 cm distance between the instrument and the infant’s head [55]. This proximity was chosen to ensure that the infant was gently enveloped by both the auditory stimulus and the subtle vibrational qualities of the instrument, while preserving a safe and non-intrusive therapeutic environment. Mothers could attend the sessions if they wished, but their presence was not a prerequisite for participation and had no bearing on study inclusion or intervention delivery, thereby maintaining both ethical and methodological neutrality. Continuous observation of the infant’s tolerance and physiological stability was maintained throughout the session to ensure safety and appropriateness of the intervention.

#### Storytelling intervention (control group)

Storytelling (ST) was implemented as an alternative live-performed intervention, serving as a non-musical, developmentally supportive stimulus for preterm infants. The initial storytelling session was conducted shortly after birth at a frequency of approximately three to four times per week. Each session lasted on average about 25 min, a duration selected to balance sufficient sensory stimulation with the limited tolerance of preterm infants.

Sessions were carried out either by the certified therapist or by a trained medical student, who positioned themselves standing or seated beside the infant to maintain a consistent and gentle presence. In cases involving multiple births (e.g., twins or triplets) placed in the same incubator or bed and allocated to the same intervention group, storytelling sessions were delivered concurrently, with attention to equal spatial positioning relative to each infant to ensure uniform exposure. Mothers were allowed to be present during the sessions; however, their presence was not required and did not influence participation in the study, preserving both ethical and methodological neutrality.

During each intervention, infants were read three selected stories from the German collection *Kuschelgeschichten zur guten Nacht* [56]: *Träum schön*,* kleiner Bär* [*Sweet Dreams*,* Little Bear*], *Wovon kleine Schwalben träumen* [*What Little Swallows Dream Of*], and *Der Igel Isidor* [*Isidor the Hedgehog*]. The narratives were delivered with a slow, controlled tempo, soft vocal quality, and rhythmic prosody to promote a soothing auditory environment. In addition, the narrator incorporated improvised modifications during and after the reading, adjusting speech rate, intonation, and phrasing in real time according to the infant’s behavioral cues and physiological responses. This responsive approach allowed for individualized, developmentally sensitive exposure to verbal and prosodic stimuli.

### Questionnaires

Evidence from previous research indicates that music therapy may have a beneficial impact on parental experiences and psychological well-being [43, 49]. To evaluate these effects, mothers in both groups, music therapy and storytelling, completed the German versions of the Center for Epidemiologic Studies Depression Scale (CES-D) and the Impact of Event Scale–Revised (IES-R). Assessments were conducted at three timepoints: prior to the first intervention session, at infants’ discharge from the hospital, and around the infant’s corrected age of one year.

#### CES-D

The Allgemeine Depressions–Skala (ADS-L) represents the German adaptation of the Center for Epidemiologic Studies Depression Scale (CES-D) [57] and is a self-report questionnaire designed to assess depressive symptomatology in adults. The scale comprises 20 self-report items formulated as first-person statements, which refer to experiences and feelings during the week preceding the assessment. The items capture a broad range of symptoms commonly associated with depression, including affective, cognitive, somatic, and social dimensions.

Responses are provided on a 4-point Likert scale, ranging from “rarely or none of the time (less than one day)” (0), “some or a little of the time (one to two days)” (1), “occasionally or a moderate amount of the time (three to four days)” (2), to “most or all of the time (five to seven days)” (3). Total scores range from 0 to 60, with higher scores indicating greater depressive symptom burden. Four items (items 4, 8, 12, and 16) are reverse scored to serve as validity checks, ensuring accurate comprehension and helping to identify potential response distortions.

#### IES-R

The Impact of Event Scale–Revised (IES-R) is a 22-item self-report instrument designed to assess subjective distress following exposure to a traumatic event. It was developed by Weiss and Marmar (1996) [58] as an expansion of the original Impact of Event Scale (IES), which was based on a theoretical framework describing the psychological consequences of traumatic stress [59]. The revision introduced 7 additional items related to the hyperarousal symptoms of post-traumatic stress disorder (PTSD), aimed at capturing an additional core dimension of the disorder. The IES-R is not a diagnostic tool, but it is widely used in both research and clinical contexts for screening and monitoring post-traumatic stress symptoms. A German version was produced through a standardized translation and back-translation process, ensuring linguistic and conceptual equivalence with the original instrument [60, 61].

The IES-R demonstrates strong psychometric properties. Its three subscales, Intrusion, Avoidance, and Hyperarousal, reflect the core symptom clusters of PTSD. The scale has shown robust construct validity, with convergent validity confirmed through structured clinical interviews and other established PTSD measures [61, 62]. Internal consistency is high, with Cronbach’s alpha values typically exceeding 0.85 [63]. Additionally, the instrument effectively distinguishes between acute stress reactions and chronic PTSD [64], providing a structured means for respondents to express psychological distress, particularly when verbal expression is difficult [19]. The IES-R has demonstrated good validity in distinguishing individuals experiencing acute stress reactions from those with post-traumatic stress disorder [64].

The subscales are composed of the following items: Intrusion (items 1, 2, 3, 6, 9, 14, 16, 20), Avoidance (items 5, 7, 8, 11, 12, 13, 17, 22), and Hyperarousal (items 4, 10, 15, 18, 19, 21). Maximum possible scores are 35 for Intrusion and Hyperarousal and 40 for Avoidance. A diagnostic index for PTSD can be calculated using the formula [60]:

  

$$\begin{aligned}X&=-0.02\times\:\mathrm{Intrusion}+0.07\times\:\mathrm{Avoidance}\\ &\quad +0.15\times\:\mathrm{Hyperarousal}-4.36\end{aligned}$$

Values above 0.0 indicate a high likelihood of PTSD. This formula has been cross-validated in two independent trauma-exposed samples, yielding reported sensitivity values between 0.70 and 0.76 and specificity values between 0.88 and 0.90.

The IES-R is appropriate for individuals who have experienced extreme and potentially traumatic events, including sexual violence, war-related experiences, natural disasters, severe accidents, life-threatening medical conditions, or highly stressful perinatal events such as preterm birth. Its structured format allows for a standardized assessment of post-traumatic stress symptoms across diverse clinical and research populations.

### Statistical analysis

Continuous variables are reported as mean with standard deviation (SD) for symmetrical distributions or normally distributed data, and as means with corresponding confidence intervals (CI) or assess the precision of an estimate. Categorical variables are presented as absolute counts and percentages. Statistical analyses and graphical representations were conducted using SAS Enterprise Guide 8.4 (SAS Institute Inc., Cary, NC, USA).

Changes across the three measurement time points (T1, T2, and T3) were compared between the two groups receiving either music therapy (MT) or storytelling (ST). Repeated measures ANOVA were not performed because the study was not designed to test a specific hypothesis regarding differences over time or between groups. Moreover, statistical significance does not necessarily correspond to clinical relevance. In line with recommendations by the American Statistical Association, p-values were therefore not used as the primary criterion for evaluating the relevance of the findings, as statistical significance alone does not provide a measure of clinical importance [62]. Instead, to provide an objective estimate of the magnitude of the observed effects, effect sizes were calculated for the between-group comparison of mean changes from baseline (T1) to follow-up at the infants’ discharge from hospital (T2) and at approximately the infants’ first birthday (T3). Effect sizes were expressed as Cohen’s *d* and calculated using pooled weighted standard deviations.

## Results

### Patients

**Fig. 1:**
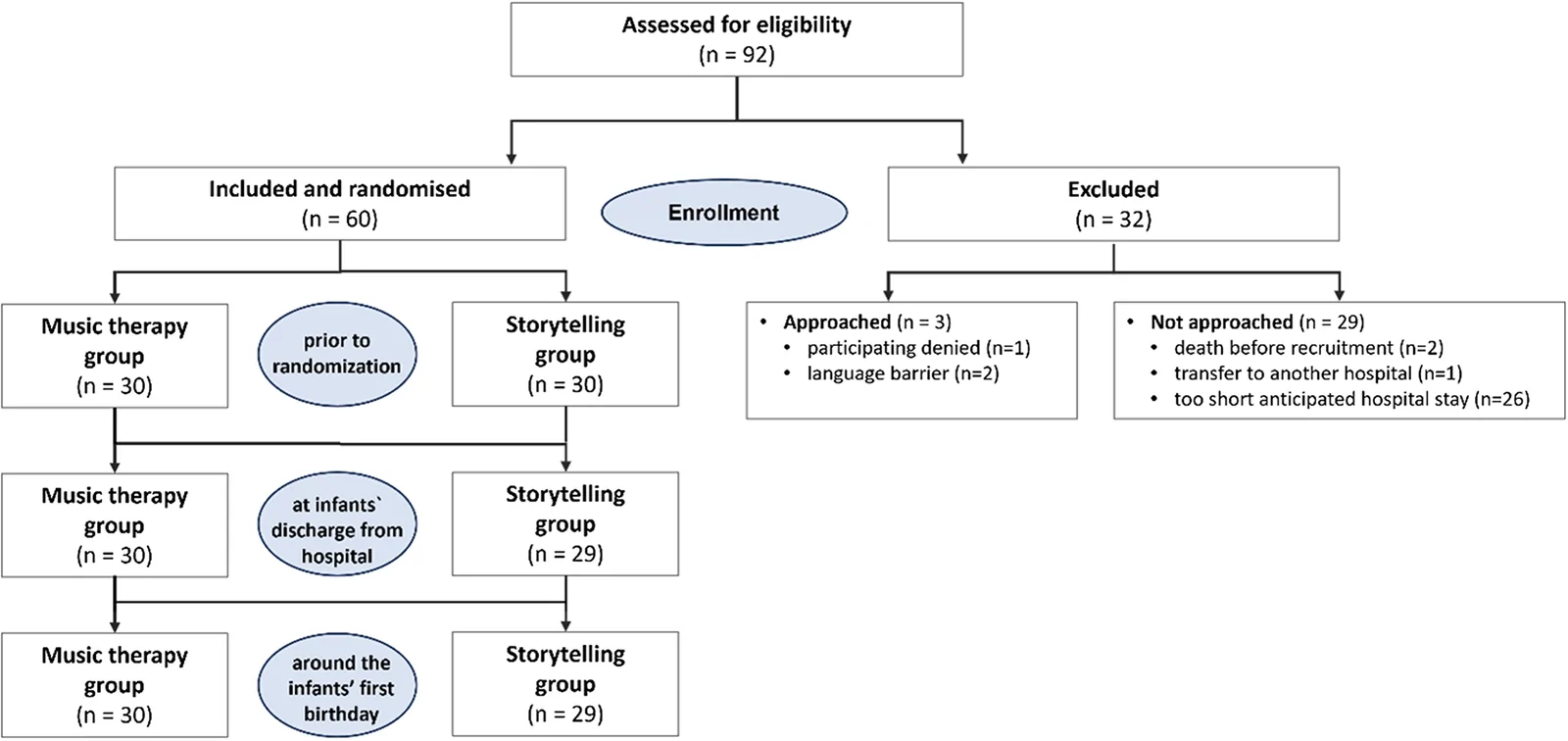
Flowchart of the included and excluded mothers of preterm infants

Between April 2023 and January 2024, a total of 60 mothers of preterm infants born at the University Hospital Essen with a gestational age of 32 + 0 to 36 + 6 weeks were considered eligible for participation in the study. An additional 32 mothers were excluded prior to enrollment. The primary reason for exclusion was an anticipated hospital stay of ≤ 7 days (*n* = 26). Other reasons included infant death prior to recruitment (*n* = 2), inability to obtain informed consent due to language barriers (*n* = 2), parental refusal to participate (*n* = 1), and transfer to another hospital (*n* = 1). The 60 enrolled participants were randomly assigned in a 1:1 ratio to one of two groups: 30 mothers whose infants received music therapy and 30 mothers whose infants received storytelling. The study enrollment and randomization process are summarized in Fig. 1 as a flowchart.

The mean ± SD gestational age was 34.6 ± 1.1 weeks (range 32 + 3–36 + 4) for MT, 34.3 ± 1.2 weeks (32 + 0–36 + 5) for ST, and 34.7 ± 1.4 weeks (32 + 0–36 + 6) among excluded participants. Maternal age at delivery was comparable between MT (33.8 ± 6.1 years, range 21–47) and ST (32.9 ± 5.3 years, range 21–45), while excluded mothers were slightly younger on average (30.8 ± 5.1 years, range 22–39). Mean gravidity was 2.9 ± 2.1 for MT and 2.5 ± 2.3 for ST, and the average number of children was 2.2 ± 1.8 for MT and 2.3 ± 1.4 for ST. Cesarean delivery was highly prevalent in all groups and occurred in all cases within the MT cohort (100%), compared with 93% in the ST cohort and 91% among excluded participants.

Overall, both groups, MT and ST, were well matched in terms of gestational age, maternal age, pregnancy history, and number of children, supporting the comparability of the groups for subsequent analyses. Minor differences, such as the slightly higher cesarean section rate in the intervention group, are acknowledged but are unlikely to substantially affect group comparability. The detailed clinical characteristics of both included and excluded mothers of preterm infants, stratified by study group, are summarized in Table 1.

**Table 1:** Clinical characteristics of the included and excluded mothers of preterm infants

	Intervention group (music therapy) (*n* = 30)	Control group (storytelling) (*n* = 30)	Excluded patients (*n* = 32)
Gestational age, weeks, mean ± SD (range)	34.6 ± 1.1 (32 + 3–36 + 4)	34.3 ± 1.2 (32 + 0–36 + 5)	34.7 ± 1.4 (32 + 0–36 + 6)
Mothers age at birth, years, mean ± SD (range)	33.8 ± 6.1 (21–47)	32.9 ± 5.3 (21–45)	30.8 ± 5.1 (22–39)
Gravida, n, mean ± SD (range)	2.9 ± 2.1 (1–9)	2.5 ± 2.3 (1–12)	2.8 ± 1.9 (1–7)
Number of children, n, mean ± SD (range)	2.2 ± 1.8 (1–10)	2.3 ± 1.4 (1–7)	2.6 ± 1.5 (1–6)
Sectio Caesarea, n (%)	30 (100)	28 (93)	29 (91)
Period between infants’ discharge and infants’ first birthday, days, mean ± SD (range)	344 ± 10.2 (340–348)	348 ± 10.2 (344–352)	357 ± 6.6 (355–359)

*Notes*. Data are presented as mean and standard deviation, if not indicated otherwise

### Interventions

A total of 552 intervention sessions were delivered, comprising 303 sessions of live music therapy and 249 sessions of storytelling. The initial MT session was delivered at a median postnatal age of 4.5 days (range 1–26), the first ST session occurred at a median age of 3 days (range 2–16). The duration of individual sessions was at median 24 min (range 20–35) for MT and 25 min (range 11–29) for ST. Infants in the MT cohort received a median of 9.5 sessions (range 1–29), while infants in the ST cohort received a median of 7 sessions (range 3–32).

### CES-D

**Fig. 2:**
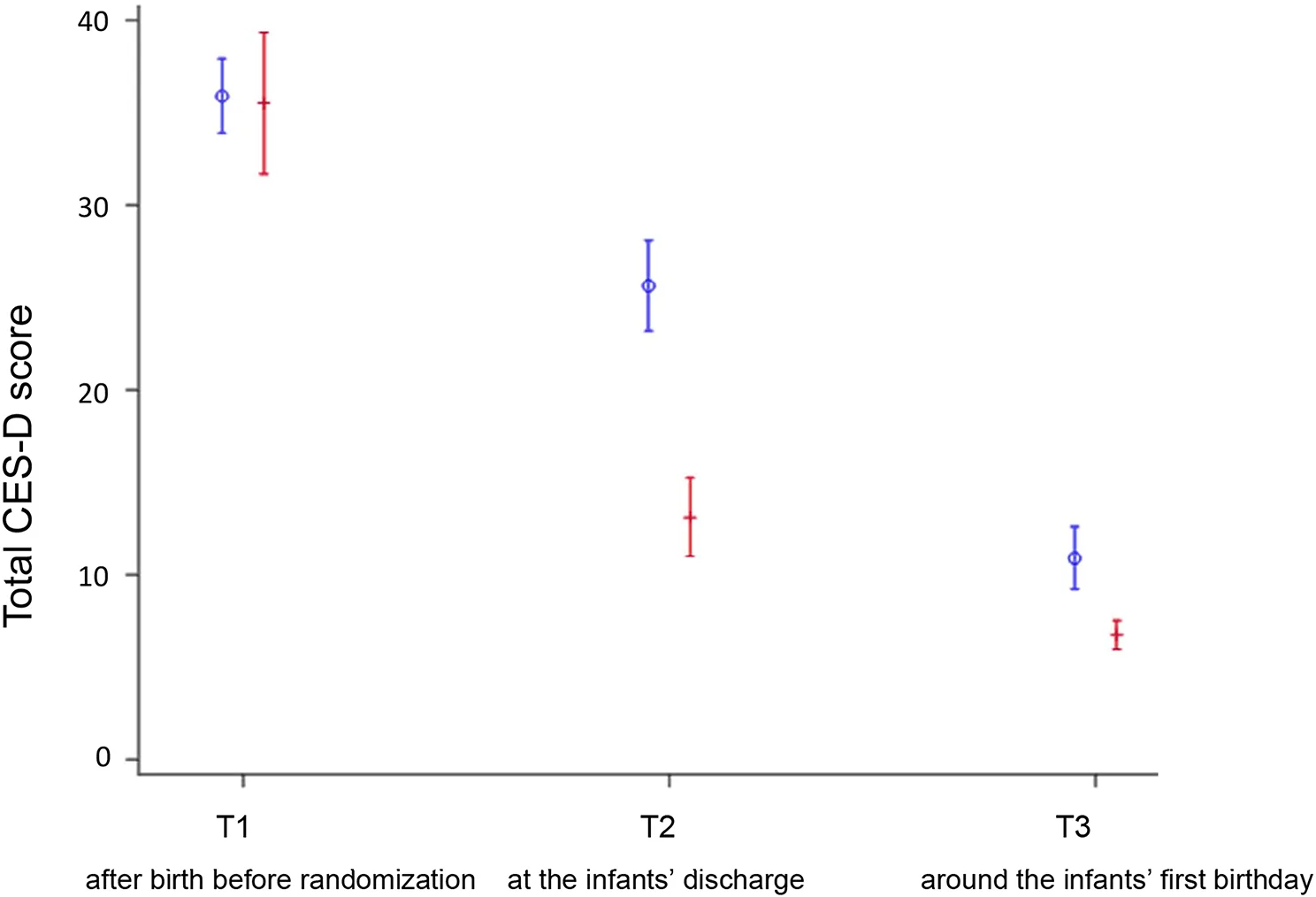
Total CES-D scores of mothers of preterm infants with music therapy (red) and storytelling (blue) directly after birth before randomization (T1), at the infants’ discharge from hospital (T2) and around the infants’ first birthday (T3). Error bars represent 95% confidence intervals

Table 2 presents the Center for Epidemiologic Studies Depression Scale (CES-D) total scores across three measurement time points (T1, T2, T3) for the two groups, music therapy and storytelling. At baseline (T1), there were no differences between mothers of infants assigned to music therapy and those assigned to storytelling with respect to depressive symptoms, as measured by the CES-D. Specifically, the mean CES-D scores were 35.5 (95% CI 31.5–39.4) for the music therapy group and 35.9 (95% CI 34.0–37.9) for the storytelling group. At T2, mothers in the music therapy intervention group exhibited lower CES-D scores (mean 13.1, 95% CI 10.9–15.3) compared with those in the control group (mean 25.2, 95% CI 22.8–27.7). At T3, both groups demonstrated further improvement. The music therapy group reached a low mean score of 6.7 (95% CI 5.9–7.6), whereas the storytelling group decreased to 10.8 (95% CI 9.1–12.4) (Table 2; Fig. 2).

**Table 2:** Total CES-D scores (mean and 95% confidence interval) of mothers of preterm infants with music therapy and storytelling directly after birth before randomization (T1), at the infants’ discharge from hospital (T2) and around the infants’ first birthday (T3)

Score	Therapy	T1		T2		T3	
		N	Mean (95% CI)	N	Mean (95% CI)	N	Mean (95% CI)
CES-D Total Score	Music therapy	30	35.5 (31.5–39.4)	30	13.1 (10.9–15.3)	30	6.7 (5.9–7.6)
	Storytelling	30	35.9 (34.0–37.9)	29	25.2 (22.8–27.7)	29	10.8 (9.1–12.4)

The mean differences of CES-D total scores from baseline (T1) to the infants’ discharge from hospital (T2) were − 22.4 (95% CI − 26.8–(–18.0)) among mothers whose infants received music therapy compared to − 10.7 (95% CI: − 13.7 to − 7.7) among mothers in the storytelling group. The mean differences of CES-D total scores from T1 to the T3 (infants’ first birthday) were − 28.8 (95% CI − 32.7–(–24.8)) among mothers whose infants received music compared to − 25.2 (95% CI − 27.7 to − 22.7) among mothers in the storytelling group. The effect sizes were strong for CES-D difference between T1 and T2 and low for the difference between T1 and T3, indicating a comparatively smaller difference between the groups at the 12-month follow-up (Table 3).Overall, both interventions were associated with a reduction in depressive symptoms from T1 to T3; however, the magnitude of improvement was higher and the rate of decline more pronounced in the music therapy group compared to the storytelling group (Table 3).

**Table 3:** Mean differences of the total CES-D Scores of mothers of preterm infants born ≥ 32 gestational weeks with music therapy (intervention group) and storytelling (control group) between assessment directly after birth before randomization (T1) and at the infants’ discharge from hospital (T2) and between assessment directly after birth before randomization (T1) and around the infants’ first birthday (T3)

	Difference between T1 and T2 Mean (95% Confidence Interval)			Difference between T1 and T3 Mean (95% Confidence Interval)		
	Intervention Group (music therapy)	Control Group (storytelling)	Effect size*	Intervention Group (music therapy)	Control Group (storytelling)	Effect size*
CES-D Total Score	–22.4 (–26.8–(–18.0))	− 10.7 (–13.7–(–7.7))	1.0	–28.8 (–32.7–(–24.8))	–25.2 (–27.7–(–22.7))	0.4

*Notes*. All data are presented as mean and 95% confidence intervals. *Cohen’s d between music therapy and storytelling group

### IES-R

**Fig. 3:**
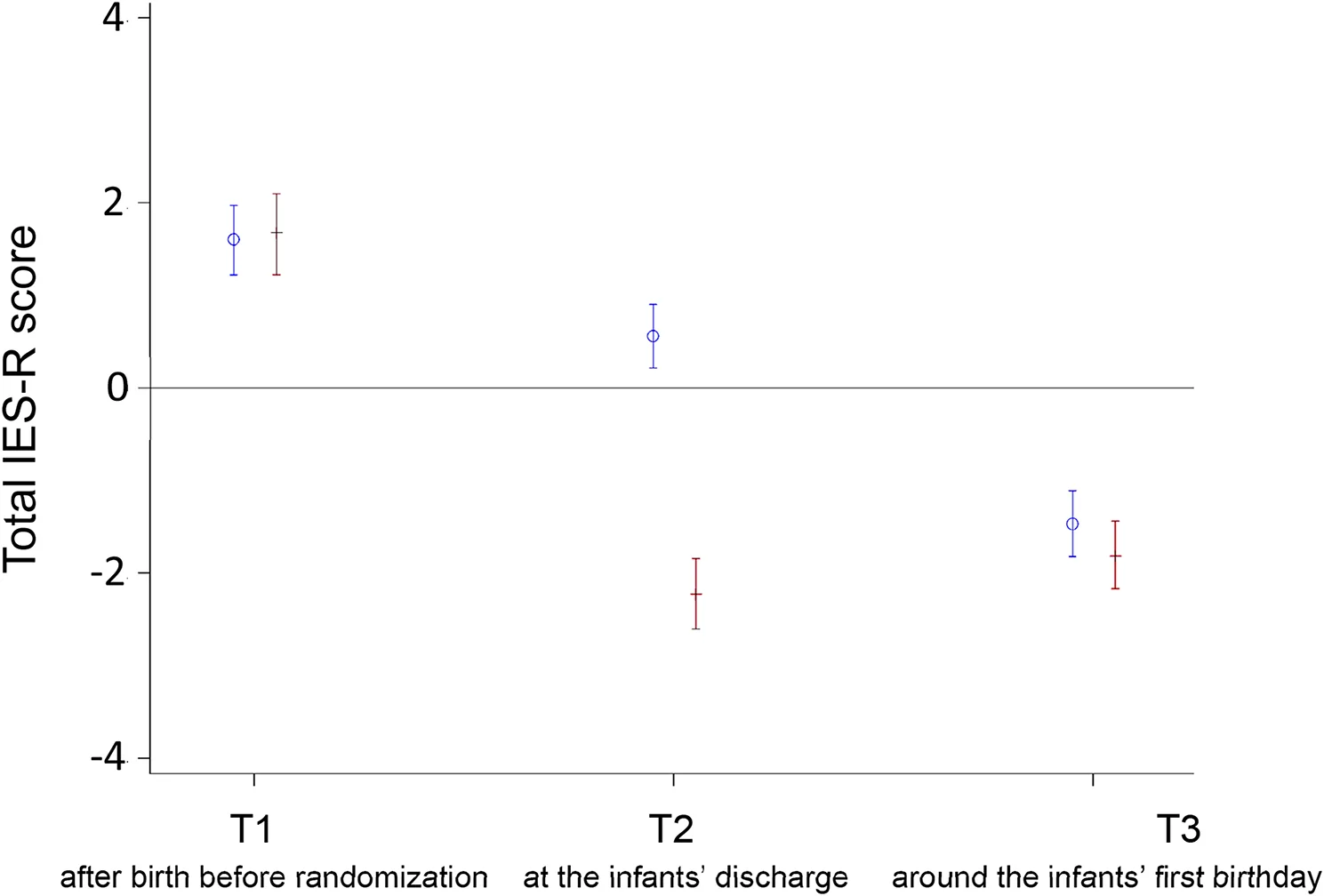
Total IES-R scores of mothers of preterm infants with music therapy (red) and storytelling (blue) directly after birth before randomization (T1), at the infants’ discharge from hospital (T2) and around the infants’ first birthday (T3). Error bars represent 95% confidence intervals

Table 4 presents the mean absolute values of the IES-R total score as well as its subscale scores (intrusion, avoidance, and hyperarousal), for mothers whose infants were assigned to either the music therapy intervention or the storytelling control intervention at T1, T2, and T3. At baseline (T1), no significant differences were observed between mothers of infants assigned to the music therapy group and those assigned to the storytelling group with regard to post-traumatic stress-related symptoms, as measured by the Impact of Event Scale–Revised (IES-R). Analysis of both, the total IES-R score and the individual subscale scores, demonstrated a more pronounced reduction from T1 to T2 in the music therapy group compared with the control group. At the one-year follow-up (T3), the observed values in the IES-R were only marginally lower than those obtained at infants’ discharge from the hospital (T2) (Table 4; Fig. 3).

**Table 4:** Total and subcategories IES-R scores (mean and 95% confidence interval) of mothers of preterm infants with music therapy and storytelling directly after birth before randomization (T1), at the infants’ discharge from hospital (T2) and around the infants’ first birthday (T3)

Score	Therapy	T1		T2		T3	
		*N*	Mean (95% CI)	*N*	Mean (95% CI)	*N*	Mean (95% CI)
IES-R Intrusion	Music therapy	30	19.9 (17.3–22.4)	30	5.5 (3.7–7.3)	30	4.7 (2.7–6.7)
	Storytelling	30	18.0 (16.2–19.7)	29	11.9 (9.9–13.8)	29	5.0 (3.5–6.4)
IES-R Avoidance	Music therapy	30	19.3 (16.9–21.7)	30	4.1 (1.9–6.3)	30	2.7 (1.0–4.4)
	Storytelling	30	19.0 (17.0–21.0)	29	11.6 (9.6–13.6)	29	4.3 (2.6–6.1)
IES-R Hyperarousal	Music therapy	30	28.8 (26.5–31.1)	30	11.5 (9.4–13.5)	30	15.3 (13.5–17.0)
	Storytelling	30	28.3 (26.7–29.9)	29	25.2 (23.5–27.0)	29	16.2 (14.4–18.1)
IES-R Total Score	Music therapy	30	1.7 (1.2–2.2)	30	–2.2 (–2.6–(–1.8))	30	–1.8 (–2–2–(–1.4)
	Storytelling	30	1.6 (1.2–1.9)	29	0.5 (0.1–0.8)	29	–1.5 (–1.9–(–1.2))

*Notes*. All data are presented as mean and 95% confidence intervals. *Cohen’s d between music therapy and storytelling group

The mean differences in IES-R total scores between T1 and T2 were − 3.9 (95% CI − 4.6–(–3.3)) in mothers with music therapy and − 1.1 (95% CI − 1.6–(–0.6)) in mothers with storytelling. All subscales, including intrusion, avoidance, and hyperarousal, demonstrated greater reductions in mothers whose infants received music therapy. The mean differences in IES-R total scores between T1 and T3 were − 3.5 (95% CI − 4.1–(–2.9)) in mothers with music therapy and − 3.1 (95% CI − 3.6–(–2.6)) in mothers with storytelling (Table 4). Effect sizes were low for IES-R and the subscales. However, strong effect sizes were observed for the overall IES-R score, and the hyperarousal subscale, while moderate effect sizes were found for the intrusion and avoidance subscales (Table 5).

**Table 5:** Mean differences of the total and subcategories IES-R Scores of mothers of preterm infants born ≥ 32 gestational weeks with music therapy (intervention group) and storytelling (control group) between assessment directly after birth before randomization (T1) and at the infants’ discharge from hospital (T2) and between assessment directly after birth before randomization (T1) and around the infants’ first birthday (T3)

	Difference between T1 and T2 Mean (95% Confidence Interval)			Difference between T1 and T3 Mean (95% Confidence Interval)		
	Intervention Group (music therapy)	Control Group (storytelling)	Effect size*	Intervention Group (music therapy)	Control Group (storytelling)	Effect size*
IES-R Intrusion	–14.4 (–17.4–(–11.3))	–6.1 (–8.7–(–3.5))	0.9	–15.2 (–18.3–(–12.0))	–13.0 (–15.3–(–10.7))	0.3
IES-R Avoidance	–15.2 (–18.4–(–12.0))	–7.4 (10.1–(–4.6))	0.9	–16.6 (–19.5–(–13.7))	–14.7 (–17.2–(–12.1))	0.3
IES-R Hyperarousal	–17.3 (–20.4–(–14.3))	–3.1 (–5.4–(–0.8))	1.6	–13.5 (–16.4–(–10.7))	–12.1 (–14.5–(–9.7))	0.1
IES-R Total Score	–3.9 (–4.6–(–3.3))	–1.1 (–1.6–(–0.6))	1.5	–3.5 (–4.1–(–2.9))	–3.1 (–3.6–(–2.6))	0.4

*Notes*. All data are presented as mean and 95% confidence intervals. *Cohen’s d between music therapy and storytelling group

## Discussion

This study investigated the effects of live music therapy compared with live storytelling on maternal psychological outcomes during and after NICU hospitalization of preterm infants. Maternal depressive and post-traumatic stress symptoms decreased substantially over time in both study groups. While baseline values were comparable between the two groups, marked divergences emerged at T2, with greater improvements observed in the music therapy group. During the infants’ first year of life, these differences attenuated, but measurable differences between the groups remained evident at T3. Large effect sizes were observed for both CES-D and IES-R at T2. Although between-group differences decreased over the first year of life, moderate effect sizes remained evident at T3, indicating sustained benefits. Importantly, the magnitude of the observed between-group differences decreased over time. While large effect sizes were observed at hospital discharge (T2), the effect sizes at the 12-month follow-up (T3) were notably smaller, although still moderate. Thus, the findings should not be interpreted as indicating an unchanged or equally strong treatment effect throughout the entire first year. Rather, the results suggest an attenuation of the initial benefit over time, with some residual advantage of music therapy remaining at 12 months. This pattern may reflect the gradual convergence of maternal psychological outcomes as families adapt to life after NICU discharge and as other experiences and sources of support increasingly influence maternal well-being. Nevertheless, the persistence of measurable between-group differences at T3 suggests that the potential effects of the intervention may extend beyond the immediate hospitalization period, even if their magnitude diminishes over time. These findings extend prior research documenting that mothers of preterm infants are at elevated risk for postpartum depression, anxiety, and post-traumatic stress symptoms [9, 65]. Meta-analyses report prevalence rates of clinically significant depressive symptoms between 25% and 40% in NICU parents, with trauma-related symptoms of similar frequency [66]. Moreover, longitudinal evidence indicates that distress may persist beyond discharge, particularly in cases of very or extremely preterm birth [66, 67]. Overall, the results are consistent with previous research demonstrating gradual psychological adjustment following preterm birth, while also indicating that clinically relevant distress may persist throughout the early postpartum period and beyond hospital discharge [68–72].

The beneficial effects of music therapy observed in this study are consistent with previous studies reporting improvements in parental stress, mood, and parent–infant interaction following family-centered music therapy interventions in neonatal settings [49, 70–72]. Notably, recent studies have demonstrated that music therapy not only reduces maternal depressive and stress symptoms during NICU hospitalization, but also positively influences maternal perception of the infant and parent–infant bonding [46, 49]. These findings support the present results, suggesting that music therapy enhances both emotional and cognitive aspects of maternal adjustment.

We found that music therapy is more effective than storytelling, particularly regarding maternal symptoms around hospital discharge and possibly in the long term, despite the relatively low intervention dose, with most infants receiving only about nine sessions.

Music therapy likely exerts its effects via multiple mechanisms, including modulation of the autonomic nervous system activity, facilitation of emotional processing, and enhancement of maternal self-efficacy and bonding [49]. These processes are particularly relevant in the NICU environment, which is frequently characterized by parental role alteration, high medical complexity, and early separation from the infant [73, 74]. In contrast, storytelling offers structured psychosocial engagement and may provide emotional support and opportunities for cognitive and relational processing. However, compared with live music therapy, it appears to engage multisensory integration processes and stress-regulatory mechanisms to a lesser extent. In particular, it lacks the combination of auditory, rhythmic, and affective stimulation inherent in live musical interaction, which may be more directly involved in modulating autonomic arousal and supporting emotional regulation.

The observed reductions in post-traumatic stress symptoms are particularly important, as preterm birth and NICU admission are commonly perceived as traumatic events [9, 65], and trauma-informed care approaches emphasize the need for early psychosocial interventions in this population [75]. Music therapy may serve as a non-verbal, emotionally regulating modality that supports trauma processing while strengthening maternal identity and perceived competence, consistent with current research demonstrating improved maternal perception and bonding [46, 49]. With cesarian section in more than 90% of mothers, all mothers were at increased risk for postpartum depression and post-traumatic stress symptoms [9, 65, 76]. While symptoms improved in both the music therapy and storytelling groups over time, music therapy demonstrated stronger short-term effects and sustained advantages even at the 12-month follow-up. These results contribute to the growing literature on maternal mental health following preterm birth [9, 65, 77] and extend prior evidence that family-centered music therapy is an effective intervention in neonatal care with measurable benefits even one year after birth [70, 71, 72]. Future research using larger randomized controlled trials is warranted to confirm these findings, clarify the underlying mechanisms (e.g., stress regulation, bonding, maternal self-efficacy). Given that the feasibility has been proven in numerous studies and settings, music therapy research should focus on facilitating integration into routine clinical care.

The transition from NICU to home represents a critical and often stressful period for parents of preterm infants, characterized by increased caregiving responsibility and reduced professional support [78, 79], with depressive and trauma-related symptoms persisting or fluctuating during the first postnatal year [70, 71, 80].

The possibility that music therapy may influence maternal outcomes even in the absence of direct maternal participation is noteworthy. In the NICU, maternal psychological well-being is closely linked to perceptions of infant safety, quality of care, and parental control. Receiving information that the infant is participating in an individualized therapeutic intervention may therefore provide reassurance and contribute to a more positive perception of the infant’s care. This may be particularly relevant for mothers who are unable to attend every intervention session because of their own medical condition, other caregiving responsibilities, or the practical circumstances of NICU hospitalization. However, because maternal expectations and perceptions of the intervention were not systematically assessed, the extent to which such indirect effects contributed to the observed outcomes remains uncertain.

Furthermore, studies have shown that early psychosocial interventions in neonatal settings can exert lasting benefits by improving parental self-efficacy and perceived competence [79]. Improvements in parental cognitive appraisal and confidence are associated with more stable long-term psychological adjustment after very preterm birth [80]. Music therapy may work through similar mechanisms by facilitating co-regulation, emotional processing, and parent–infant bonding during a period of heightened vulnerability [81].

The clinical relevance of these findings warrants particular consideration beyond the observed differences between groups. The large effect sizes observed for both CES-D and IES-R at T2 indicate that the reductions in depressive and post-traumatic stress symptoms were substantial in magnitude. The persistence of moderate effect sizes at T3 further suggests that the potential benefit of music therapy may extend beyond the immediate NICU period and remain relevant during the transition to home and the first year of parenthood. This is clinically important given that persistent depressive and trauma-related symptoms may adversely affect maternal well-being, parental self-efficacy, and the developing parent–infant relationship.

However, the observed changes should be interpreted with caution with regard to established clinical thresholds. The present study was not designed to determine diagnostic remission or to assess whether individual participants crossed established thresholds for clinically significant symptomatology, and minimally clinically important differences (MCIDs) for CES-D and IES-R changes specifically in mothers of preterm infants receiving NICU-based interventions have not been established sufficiently to allow a direct interpretation of the present findings. Consequently, the effect sizes cannot be equated directly with clinically meaningful improvement at the individual patient level. Nevertheless, the combination of substantial reductions in symptom scores, large between-group effects at hospital discharge, and sustained moderate effects at 12 months provides evidence of a potentially meaningful clinical benefit. Future studies should therefore complement continuous symptom measures with clinically interpretable outcomes, such as the proportion of mothers moving below validated clinical cut-offs or achieving a predefined clinically meaningful improvement, to better determine the clinical impact of music therapy.

Some methodological limitations should be considered. Potential attention and nonspecific therapeutic effects should also be considered when interpreting the observed differences between the two intervention groups. Both groups received structured therapeutic interaction, including regular professional attention, emotional support, and opportunities for communication and relational engagement. Therefore, some of the observed improvements in maternal depressive and post-traumatic stress symptoms may be attributable to nonspecific factors common to both interventions, such as increased professional attention, perceived support, therapeutic alliance, or positive expectations regarding the intervention. These factors may have contributed to the overall improvement observed in both groups and may partly explain why symptom reductions were also evident in the storytelling group. However, the greater improvements observed in the music therapy group suggest that, in addition to these shared therapeutic factors, music therapy may provide intervention-specific benefits. Nevertheless, the present study design does not allow the effects of music therapy to be completely separated from attention, expectancy, and other nonspecific therapeutic effects. Future trials should therefore consider additional control conditions, such as usual care or attention-matched non-therapeutic interventions, as well as the assessment of treatment expectations and therapeutic alliance, to better distinguish intervention-specific effects from general effects of therapeutic attention and support.

Participants could not be blinded to group allocation because of the inherently perceptible nature of the interventions. Knowledge of receiving music therapy or storytelling may therefore have influenced mothers` expectations and self-reported outcomes. Such expectancy effects are particularly relevant in behavioral and psychosocial interventions, in which treatment preferences and perceived credibility may contribute to observed improvements. Future trials should therefore consider assessing treatment expectations and preferences at baseline and, where feasible, therapeutic alliance and perceived treatment credibility, to better account for these potential sources of bias. Another limitation concerns the limited number and timing of assessment points, which do not allow the longitudinal trajectories of maternal post-traumatic stress symptoms to be adequately characterized. Previous research suggests that individuals exposed to potentially traumatic events may follow different symptom trajectories over time, including resilience, recovery, delayed-onset post-traumatic stress symptoms, and chronic or persistent symptoms. With assessments at baseline, hospital discharge of the infant, and again at the age of 12 months, the present study could not determine which of these trajectories individual mothers followed. Future studies should include more frequent assessments, particularly during the transition from hospital to home and throughout the first postnatal year, to better characterize the temporal course of maternal post-traumatic stress symptoms and identify distinct symptom trajectories. Another limitation is the single-center design of the study, which may limit the generalizability of the findings to other neonatal care settings, healthcare systems, and populations. Characteristics of the participating NICU, including its organizational structure, staffing, family-centered care practices, and availability of psychosocial support, may have influenced both the feasibility and the effects of the interventions. In addition, the characteristics of the participating mothers and infants may not fully represent the broader population of families experiencing preterm birth. Consequently, the observed effects should be interpreted cautiously with regard to their applicability to other clinical contexts. Multicenter studies involving more diverse patient populations and different neonatal care settings are needed to assess the external validity and transferability of these findings.

Furthermore, it would be advisable to extend the interventions beyond the duration of the hospital stay and to investigate this effectiveness. Maternal outcomes were assessed exclusively via self-report instruments, although validated, these measures may be influenced by response bias.

An additional limitation is that several factors that may have influenced maternal psychological outcomes during the follow-up period were not systematically assessed. Maternal attendance at individual music therapy or storytelling sessions was not recorded, and therefore the extent of mothers’ direct exposure to the respective interventions could not be quantified. Furthermore, information on other forms of mental health support or psychological treatment received by mothers during the follow-up period was not collected. Finally, we did not assess whether mothers continued to engage in music- or storytelling-related activities after discharge from the neonatal unit. These factors may have contributed to maternal psychological outcomes and cannot be ruled out as potential confounders, particularly at the approximately one-year follow-up. Future studies should systematically document maternal attendance, subsequent mental health interventions, and continuation of intervention-related activities after discharge to better characterize intervention exposure and potential confounding over the follow-up period.

## Conclusion

In summary, this study suggests that music therapy during the hospitalization of preterm infants is associated with long-lasting improvements in maternal psychological well-being and reductions in maternal depression and psychological distress. Music therapy demonstrated larger short-term effects compared with storytelling and sustained advantages through the infants’ first birthday. These findings support the integration of family-centered music therapy as a meaningful adjunct intervention in neonatal intensive care settings and highlight the importance of early psychosocial support in promoting long-term maternal mental health following preterm birth. Larger, multicenter randomized controlled trials are warranted to confirm the observed effects, assess their generalizability, and further determine the long-term clinical relevance of music therapy for maternal mental health following preterm birth.

## Data Availability

All data generated or analyzed during this study are included in this article. Further enquiries can be directed to the corresponding author.
